# Association of Mortality and Years of Potential Life Lost With Active Tuberculosis in the United States

**DOI:** 10.1001/jamanetworkopen.2020.14481

**Published:** 2020-09-23

**Authors:** Christian Lee-Rodriguez, Paul Y. Wada, Yun-Yi Hung, Jacek Skarbinski

**Affiliations:** 1 Internal Medicine Residency Program, Oakland Medical Center, Kaiser Permanente Northern California, Oakland; 2 Division of Research, Kaiser Permanente Northern California, Oakland; 3 Department of Infectious Diseases, Oakland Medical Center, Kaiser Permanente Northern California, Oakland

## Abstract

**Importance:**

Active tuberculosis (TB) disease leads to substantial mortality but is preventable through screening and treatment for latent TB infection. Early mortality after TB diagnosis (≤1 year) is well described, but delayed mortality (>1 year) among patients with active TB is poorly understood.

**Objective:**

To compare early and delayed mortality and years of potential life (YPL) lost among patients with active TB disease vs an age-, sex-, and year of diagnosis–matched comparison cohort without active TB disease.

**Design, Setting, and Participants:**

This retrospective cohort study, conducted in the integrated health system of Kaiser Permanente Northern California, included patients with microbiologically confirmed active TB disease from January 1, 1997, to December 31, 2017, and a control cohort matched by age, sex, and year of diagnosis. Multivariable models were used to adjust for demographic and clinical characteristics. Patients with active TB disease prior to 1997 were excluded. Data were analyzed from January 1, 2019, to January 31, 2020.

**Exposure:**

Microbiologically confirmed TB disease.

**Main Outcomes and Measures:**

Early (≤1 year after TB diagnosis) and delayed (>1 year after TB diagnosis) all-cause mortality.

**Results:**

A total of 2522 patients who had active TB from 1997 to 2017 were identified, with 17 166 person-years of follow-up. The comparison cohort included 100 880 persons with 735 726 person-years of follow-up. In the active TB and comparison cohorts, similar percentages of persons were male (56.3% vs 55.6%), aged 45 to 64 years (33.7% vs 33.7%), and aged 65 years or older (24.7% vs 24.7%). Both early mortality (7.0%) and delayed mortality (16.3%) were higher among patients with active TB disease compared with those without active TB disease (1.1% and 12.0%, respectively). Patients with active TB disease had a significantly higher risk for early (adjusted hazard ratio [aHR], 7.29; 95% CI, 6.08-8.73) and delayed (aHR, 1.78; 95% CI, 1.61-1.98) mortality compared with the comparison cohort (*P* < .001). Active TB disease was associated with an adjusted −7.0 (95% CI, −8.4 to −5.5) YPL lost compared with the comparison cohort.

**Conclusions and Relevance:**

In this study, patients with active TB disease had significantly higher early and delayed all-cause mortality when adjusting for demographic and clinical characteristics. These findings suggest that TB prevention through screening and treatment of latent TB infection could reduce mortality and YPL lost due to active TB disease.

## Key Points

**Question:**

Do patients with active tuberculosis disease have an increased risk of delayed mortality (ie, more than 1 year after tuberculosis diagnosis)?

**Findings:**

In this cohort study, 2522 patients with active tuberculosis disease had a 78% increased risk of death more than 1 year after tuberculosis diagnosis compared with a matched comparison cohort of 100 880 individuals without active tuberculosis.

**Meaning:**

These findings suggest that the mortality risk and longevity loss associated with active tuberculosis disease is underappreciated; patients with active tuberculosis disease appear to have a long-term increased risk of mortality.

## Teaser

This cohort study examines the risk of mortality and years of potential life lost among patients with active tuberculosis disease compared with individuals without active tuberculosis.

## Introduction

In low-incidence countries, such as the United States, most active tuberculosis (TB) disease is due to reactivation of latent TB infection (LTBI)^1^ and thus is preventable through screening for and treatment of LTBI.^2^ However, despite LTBI screening being an established recommendation by numerous guidelines,^2^ many studies have documented low screening and treatment rates.^3^ For example, in California, only 12% of the estimated 2 million people with LTBI have received LTBI treatment.^4^ As active TB incidence has declined in the United States, the perceived value of TB prevention has declined for clinicians and health systems. As we have shown previously, active TB disease continues to be associated with high health care use and mortality within the first year after diagnosis.^5^ Many studies have documented early mortality in the first year after TB diagnosis,^1^ but few have explored delayed mortality or residual mortality risk more than 1 year after TB diagnosis.^6,7^ Recognizing delayed mortality risk is important to understanding the full burden of active TB disease and the potential value of LTBI screening and treatment programs.

Persons who develop active TB disease are at high risk of mortality in the first year after diagnosis, yet those who survive their initial episode of active TB disease are considered cured and are not expected to have increased residual mortality. Some models of cost-effectiveness rely on this assumption.^8^ Unfortunately, health deficits might persist even after successful treatment and lead to increased risk of death.^9,10,11,12,13,14,15,16,17,18,19,20,21^ Thus far, only 1 study in the United States has assessed delayed mortality and years of potential life (YPL) lost among TB survivors and has shown an increased risk of mortality and YPL lost among TB survivors compared with patients with LTBI.^6,7^ Smaller studies from the Netherlands; Estonia; Liverpool, the United Kingdom; and Barcelona, Spain have also found increased mortality among persons with active TB disease.^22,23,24,25^ However, all of the studies used different methods, and only 2 studies addressed delayed mortality among TB survivors.^22,24^

The present study expands on previous work as we (1) assess the risks of early and delayed mortality in a single, large retrospective cohort and thus provide data on the relative contributions of early vs delayed mortality; (2) assess the possible association of different risk factors with early vs delayed mortality; (3) compare the incidence of early and delayed mortality among patients with active TB compared with a matched cohort and adjust for key comorbidities to ascertain whether excess mortality risk among patients with active TB disease is due to active TB disease or underlying risk factors that predispose patients to develop active TB disease; and (4) estimate YPL gained or lost among patients with active TB disease compared with a comparison cohort adjusting for demographic and clinical characteristics.

## Methods

This cohort study was approved by the Kaiser Permanente Northern California (KPNC) Institutional Board Review with a waiver of the requirement for informed consent as a data-only study using information collected as part of routine care. The study was supported by the KPNC Graduate Medical Education Program, Kaiser Foundation Hospitals, and the KPNC Community Benefit Program and followed the Strengthening the Reporting of Observational Studies in Epidemiology (STROBE) reporting guideline.

Kaiser Permanente Northern California is part of a large, integrated health system and provides all health care services to approximately 4.4 million members in Northern California. In this retrospective cohort study, we included all patients diagnosed with microbiologically-confirmed active TB in KPNC between 1997 and 2017. Data were extracted from KPNC administrative databases and from an integrated electronic health records (EHR) databases (Epic). All patients with at least 1 culture or polymerase chain reaction test positive for *Mycobacterium tuberculosis* between January 1, 1997, and December 31, 2017, were identified and included in the study. Patients were excluded if they had active TB disease prior to 1997. In addition, patients had to be KPNC members with at least 1 month of membership prior to TB diagnosis, membership at the time of TB diagnosis, or membership for at least 1 month in the 1 year after TB diagnosis. The active TB cohort was compared with a non-TB comparison cohort matched 40:1 for age, sex, and year of diagnosis.

The primary outcome was death from any cause. Death dates for all patients were obtained from KPNC databases, the California Death Certificate file, and the Social Security Death Master file. Follow-up time was calculated from the date of active TB diagnosis in the active TB cohort or midyear in the comparison group (July 1) until date of death or end of the follow-up period on December 31, 2018. Early mortality was defined as death in the first year (≤1 year) after diagnosis or match date, and delayed mortality was defined as death more than 1 year after diagnosis or match date.

Age, sex, and race/ethnicity data were extracted from demographic fields within administrative and EHR databases. Kaiser Permanente Northern California maintains validated disease registries for patients living with diabetes, HIV infection, and end-stage kidney disease for research purposes and clinical management; all new diagnoses are reviewed monthly and verified prior to inclusion in the registry. History of solid organ transplantation was obtained from the EHR using *International Classification of Diseases, Ninth Revision (ICD-9) *and *International Statistical Classification of Diseases and Related Health Problems, Tenth Revision (ICD-10)* diagnosis codes. History of cancer diagnosis was based on Surveillance, Epidemiology, and End Results Program codes that are routinely assessed for all KPNC members with malignancy. Site of active TB disease was extracted from laboratory databases based on source of specimens positive for *Mycobacterium tuberculosis* and classified as pulmonary (sputum, bronchoalveolar lavage, lung biopsy), extrapulmonary, or both. Data on specimen source were unavailable prior to July 2002.

### Statistical Analysis

Data were analyzed from January 2019 to January 2020. All analyses were performed using SAS, version 9.4 (SAS Institute Inc). Bivariate and multivariable analyses were conducted to examine the relationships between patients’ demographic and clinical characteristics and death. Clinically relevant and statistically significant variables in the bivariate analysis were included in a multivariable Cox proportional hazards model to assess factors associated with early and delayed mortality among patients with active TB disease. Similarly, clinically relevant and statistically significant variables in the bivariate analysis were included in a multivariable Cox proportional hazards model to estimate adjusted incidence rates for early and late mortality among patients with active TB disease and among the comparison cohort. Adjusted incidence rate ratios were calculated to estimate differences between patients with active TB disease and the comparison cohort. The Kaplan-Meier method was used to calculate the survival curves for patients with active TB disease and the comparison cohort.

We calculated YPL lost or gained for both the active TB and comparison cohorts compared with the United States Life Tables, 2015.^26^ The YPL represents the potential life expectancy for each individual, and differences in the average YPL between the active TB and comparison cohorts represent potential disparities in life expectancy between the 2 groups. We adapted the life expectancy method^27^ to allow for both loss and gain of life expectancy, as the United States Life Tables do not accurately reflect life expectancy in California as a whole or at KPNC; both have higher than average life expectancy, and thus the standard life expectancy method based on US standardized tables underestimates YPL lost. The YPL was calculated as the difference between the observed age at death, end of membership, or end of follow-up and the expected sex- and age-adjusted life expectancy based on the United States Life Tables, 2015^26^; this value could be either a loss (died before average life expectancy) or a gain (died after average life expectancy). We then conducted linear regression modeling to estimate the differences in YPL between the active TB and comparison cohorts adjusting for demographic variables and comorbidities. A 2-sided *P* < .05 was considered statistically significant, and 95% CIs were presented for all model estimates.

## Results

We identified 2522 persons with 17 166 persons-years of follow-up who had microbiologically confirmed active TB disease between 1997 and 2017 and included 100 880 persons with 735 726 person-years of follow-up in the comparison cohort. The active TB relapse rate in KPNC from 1997 to 2017 was low, with 8 of 2522 active TB patients experiencing relapse in 17 166 person-years of follow-up, for a rate of 0.047 per 100 person-years. In the active TB and comparison cohorts, similar percentages of persons were male (56.3% vs 55.6%), aged 45 to 64 years (33.7% vs 33.7%), and aged 65 years or older (24.7% vs 24.7%) (Table 1). The 2 groups had notably different race/ethnicity distributions: in the active TB cohort, 57.4% were of Asian/Pacific Islander race/ethnicity compared with 14.2% of the comparison cohort. In the active TB cohort, 7.0% had early mortality and 16.3% had delayed mortality compared with 1.1% and 12.0% in the comparison cohort, respectively. Overall survival probability was significantly lower (log-rank *P* < .001) in the active TB cohort compared with the comparison cohort (Figure). Compared with the comparison cohort, persons in the active TB group were more likely to have diabetes (25.5% vs 9.4%), HIV infection (2.5% vs 0.3%), end-stage kidney disease (2.5% vs 0.3%), prior solid organ transplantation (0.8% vs 0.1%), and history of cancer diagnosis (7.3% vs 4.6%) (*P* < .001). Overall, 34.1% of the active TB cohort had at least 1 comorbidity compared with 13.3% of the comparison cohort (*P* < .001).

**Table 1.** Characteristics of Persons With Active TB Disease and an Age-, Sex-, and Date of Diagnosis–Matched Cohort of Patients Without Active TB Disease Stratified by Survival Status in a Large, Integrated Health System, Kaiser Permanente Northern California, 1997-2017

Characteristic	Cohort, No. (%)								
	Active TB				Comparison				
	All	Living	Died		All	Living	Died	
			In year 1	After year 1			In year 1	After year 1
Total	2522 (100)	1935 (76.7)	176 (7.0)	411 (16.3)	100 880 (100)	87 721 (87.0)	1070 (1.1)	12 089 (12.0)
Sex								
Male	1420 (56.3)	1031 (53.3)	114 (64.8)	275 (66.9)	56 088 (55.6)	47 568 (54.2)	706 (66.0)	7814 (64.6)
Female	1102 (43.7)	904 (46.7)	62 (35.2)	136 (33.1)	44 792 (44.4)	40 153 (45.8)	364 (34.0)	4275 (35.4)
Age, y								
0-24	231 (9.2)	228 (11.8)	2 (1.1)	1 (0.2)	9240 (9.2)	9176 (10.5)	5 (0.5)	59 (0.5)
25-44	820 (32.5)	789 (40.8)	9 (5.1)	22 (5.4)	32 800 (32.5)	32 249 (36.8)	24 (2.2)	527 (4.4)
45-64	849 (33.7)	656 (33.9)	46 (26.1)	147 (35.8)	33 960 (33.7)	31 352 (35.7)	154 (14.4)	2454 (20.3)
≥65	622 (24.7)	262 (13.5)	119 (67.6)	241 (58.6)	24 880 (24.7)	14 944 (17.0)	887 (82.9)	9049 (74.9)
Race/ethnicity^a^								
Non-Hispanic								
White	242 (9.6)	126 (6.5)	34 (19.3)	82 (20.0)	50 201 (49.8)	41 176 (46.9)	759 (70.9)	8266 (68.4)
Black	181 (7.2)	131 (6.8)	23 (13.1)	27 (6.6)	6849 (6.8)	5840 (6.7)	85 (7.9)	924 (7.6)
Hispanic	367 (14.6)	290 (15.0)	26 (14.8)	51 (12.4)	15 110 (15.0)	13 871 (15.8)	81 (7.6)	1158 (9.6)
Asian/Pacific Islander	1448 (57.4)	1156 (59.7)	88 (50.0)	204 (49.6)	14 293 (14.2)	13 227 (15.1)	93 (8.7)	973 (8.0)
American Indian/Alaska Native/other/mixed race	284 (11.3)	232 (12.0)	5 (2.8)	47 (11.4)	14 427 (14.3)	13 607 (15.5)	52 (4.9)	768 (6.4)
Comorbidities^a^								
Diabetes	643 (25.5)	402 (20.8)	56 (31.8)	185 (45.0)	9476 (9.4)	6623 (7.6)	293 (27.4)	2560 (21.2)
End-stage kidney disease	63 (2.5)	16 (0.8)	19 (10.8)	28 (6.8)	257 (0.3)	112 (0.1)	34 (3.2)	111 (0.9)
HIV infection	64 (2.5)	46 (2.4)	9 (5.1)	9 (2.2)	260 (0.3)	216 (0.2)	3 (0.3)	41 (0.3)
Prior solid organ transplant	19 (0.8)	10 (0.5)	4 (2.3)	5 (1.2)	84 (0.1)	51 (0.1)	5 (0.5)	28 (0.2)
History of cancer diagnosis	183 (7.3)	64 (3.3)	57 (32.4)	62 (15.1)	4593 (4.6)	2737 (3.1)	297 (27.8)	1559 (12.9)
Active TB site at diagnosis								
Pulmonary and extrapulmonary	173 (6.9)	124 (6.4)	19 (10.8)	30 (7.3)	NA	NA	NA	NA
Pulmonary only	1216 (48.2)	970 (50.1)	80 (45.5)	166 (40.4)	NA	NA	NA	NA
Extrapulmonary only	452 (17.9)	381 (19.7)	21 (11.9)	50 (12.2)	NA	NA	NA	NA
Data not available prior to July 2002	681 (27.0)	460 (23.8)	56 (31.8)	165 (40.1)	NA	NA	NA	NA

Abbreviations: NA, not applicable; TB, tuberculosis.

^a^ Race/ethnicity and presence of selected comorbidities significantly different between active TB and comparison cohort for all categories (*P* < .001).

**Figure.**
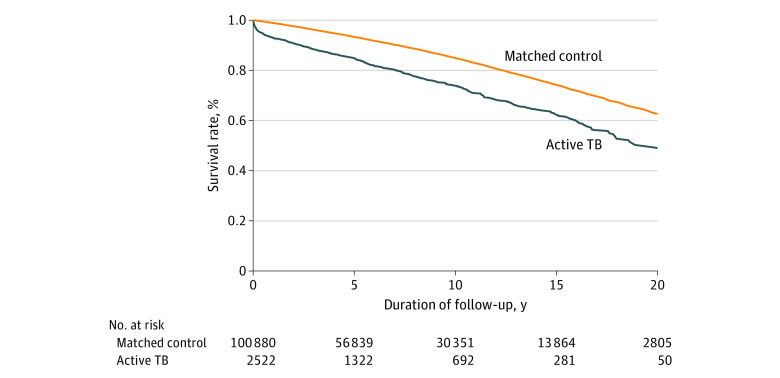
Survival Probability Among Patients With Active Tuberculosis (TB) Disease Compared With an Age-, Sex-, and Date of Diagnosis–Matched Cohort of Patients Without Active TB Disease, Kaiser Permanente Northern California, 1997-2017

Using a multivariable Cox proportional hazards model, we assessed risk factors for early and delayed mortality among patients with active TB disease. We found that age group (0-24 years [adjusted hazard ratio {aHR}, 0.06 {95% CI, 0.01-0.23}; *P* < .001], 25-44 years [0.06 {95% CI, 0.03-0.13}; *P* < .001], and 45-64 years [aHR, 0.28 {95% CI, 0.20-0.41}; *P* < .001] vs ≥65 years) was significantly associated with lower risk of early mortality; persons of Black, non-Hispanic race/ethnicity (aHR, 2.06 [95% CI, 1.18-3.61]; *P* = .01), those with comorbidities (end-stage kidney disease [aHR, 3.91 {95% CI, 2.33-6.58}; *P* < .001], HIV infection [aHR, 4.07 {95% CI, 1.93-8.58}; *P* < .001], prior solid organ transplant [aHR, 5.14 {95% CI, 1.79-14.75}; *P* = .002], or history of cancer [aHR, 3.86 {95% CI, 2.76-5.40}; *P* < .001]), and those with pulmonary and extrapulmonary TB (aHR, 2.08 [95% CI, 1.10-3.92]; *P* = .02) were all significantly more likely to have early mortality (Table 2). We found lower risk of delayed mortality by age group (0-24 years [aHR, 0.01 {95% CI, 0.002-0.09}; *P* < .001], 25-44 years [aHR, 0.06 {95% CI, 0.04-0.10}; *P* < .001], and 45-64 years [aHR, 0.29 {95% CI, 0.23-0.36}; *P* < .001] vs ≥65 years) and race/ethnicity (Hispanic [aHR, 0.62 {95% CI, 0.43-0.89}; *P* = .01] and Asian/Pacific Islander [aHR, 0.58 {95% CI, 0.44-0.77}; *P* < .001) vs White, non-Hispanic) but higher risk of delayed mortality among men (aHR, 1.33 [95% CI, 1.07-1.64]; *P* = .01), persons with comorbidities (diabetes [aHR, 1.67 {95% CI, 1.36-2.06}; *P* < .001], end-stage kidney disease [aHR, 4.02 {95% CI, 2.70-5.98}; *P* < .001], prior solid organ transplantation [aHR, 2.86 {95% CI, 1.15-7.13}; *P* < .001], or history of cancer [aHR, 1.77 {95% CI, 1.34-2.35}; *P* < .001]), and those with pulmonary and extrapulmonary TB (aHR, 1.67 [95% CI, 1.05-2.64]; *P* = .03).

**Table 2.** Factors Associated With Early and Delayed Mortality Among Patients With Active TB Disease, Kaiser Permanente Northern California, 1997-2017 (N = 2522)^a^

Variable	Mortality	
	aHR (95% CI)	*P* value
**Early**		
Sex		
Male	1.06 (0.77-1.47)	.71
Female	1 [Reference]	
Age, y		
0-24	0.06 (0.01-0.23)	<.001
25-44	0.06 (0.03-0.13)	<.001
45-64	0.28 (0.20-0.41)	<.001
≥65	1 [Reference]	
Race/ethnicity		
Non-Hispanic		
White	1 [Reference]	
Black	2.06 (1.18-3.61)	.01
Hispanic	1.14 (0.66-1.95)	.64
Asian/Pacific Islander	0.92 (0.60-1.42)	.71
American Indian/Alaska Native/other/mixed race	0.30 (0.12-0.78)	.01
Comorbidities		
Diabetes	0.80 (0.56-1.13)	.20
End-stage kidney disease	3.91 (2.33-6.58)	<.001
HIV infection	4.07 (1.93-8.58)	<.001
Prior solid organ transplant	5.14 (1.79-14.75)	.002
History of cancer diagnosis	3.86 (2.76-5.40)	<.001
Active TB site at diagnosis		
Pulmonary only	1.60 (0.97-2.65)	.07
Pulmonary and extrapulmonary	2.08 (1.10-3.92)	.02
Extrapulmonary only	1 [Reference]	
Data not available prior to July 2002	2.21 (1.32-3.70)	.003
**Delayed**		
Sex		
Male	1.33 (1.07-1.64)	.01
Female	1 [Reference]	
Age, y		
0-24	0.01 (0.002-0.09)	<.001
25-44	0.06 (0.04-0.10)	<.001
45-64	0.29 (0.23-0.36)	<.001
≥65	1 [Reference]	
Race/ethnicity		
Non-Hispanic		
White	1 [Reference]	
Black	0.74 (0.47-1.17)	.20
Hispanic	0.62 (0.43-0.89)	.01
Asian/Pacific Islander	0.58 (0.44-0.77)	<.001
American Indian/Alaska Native/other/mixed race	0.80 (0.55-1.16)	.23
Comorbidities		
Diabetes	1.67 (1.36-2.06)	<.001
End-stage kidney disease	4.02 (2.70-5.98)	<.001
HIV infection	1.46 (0.73-2.92)	.29
Prior solid organ transplant	2.86 (1.15-7.13)	.02
History of cancer diagnosis	1.77 (1.34-2.35)	<.001
Active TB site at diagnosis		
Pulmonary only	1.32 (0.95-1.83)	.10
Pulmonary and extrapulmonary	1.67 (1.05-2.64)	.03
Extrapulmonary only	1 [Reference]	
Data not available prior to July 2002	1.34 (0.96-1.87)	.08

Abbreviations: aHR, adjusted hazard ratio; TB, tuberculosis.

^a^ Cox proportional hazards model used. The comparison group for both analyses is those who survived. This includes only persons with active TB disease. Adjusted models include all variables that are significant in the univariate analysis.

We used multivariable Cox proportional hazards models to compare the incidence of death between the active TB and comparison cohorts and estimated the death incidence rates due to early and delayed mortality by cohorts’ demographics characteristics and by each of the comorbidities (eTable in the Supplement). The adjusted mortality rate among persons in the active TB cohort was 24.31 (95% CI, 21.98-26.64) per 1000 person-years compared with 13.44 (95% CI, 13.17-13.70) for persons in the active TB cohort; the adjusted rate ratio was 1.81 (95% CI, 1.63-1.99) (*P* < .001). Table 3 presents aHRs between the active TB and the comparison cohort. Mortality probability was higher among TB patients compared with the comparison cohort for both within year 1 after diagnosis or matching date (early mortality) and after year 1 (delayed mortality). The adjusted risks of early mortality (aHR, 7.29; 95% CI, 6.08-8.73; *P* < .001) and delayed mortality (aHR, 1.78; 95% CI, 1.61-1.98; *P* < .001) were significantly higher among patients with active TB disease compared with the comparison cohort after adjusting for age, sex, race/ethnicity, and selected comorbidities. Thus, on average, even 1 year after diagnosis, TB exposure was associated with a 78% increase in mortality over the follow-up period after controlling for age, sex, race/ethnicity, and selected comorbidities. The adjusted mortality rate was significantly higher among all subgroups (except persons aged 0-24 years) with active TB disease compared to the comparison cohort even after adjusting for demographic characteristics and comorbidities. Persons in the active TB cohort had an adjusted difference in YPL of −7.0 (95% CI, −8.4 to −5.5) years compared with those in the comparison cohort (*P* < .001) (Table 4). Patients in the active TB cohort had a significant YPL loss compared with the comparison cohort among all subgroups except persons aged 0-24 years (3.9 [95% CI, −8.4 to 16.2] years; *P* = .26) and those aged 25-44 years (−2.0 [95% CI, −4.5 to 1.5] years; *P* = .15).

**Table 3.** Cox Proportional Hazard Model Estimates of Early and Delayed Mortality Risk Among Patients With Active TB Disease Compared With an Age-, Sex-, and Date of Diagnosis–Matched Cohort of Patients Without Active TB Disease Adjusting for Demographic Characteristics And Selected Comorbidities, Kaiser Permanente Northern California, 1997-2017^a^

Variable	Mortality	
	aHR (95% CI)	*P* value
**Early**		
Cohort		
Active TB	7.29 (6.08-8.73)	<.001
Comparison	1 [Reference]	
Sex		
Male	1.11 (0.99-1.25)	.08
Female	1 [Reference]	
Age, y		
0-24	0.03 (0.02-0.07)	<.001
25-44	0.04 (0.03-0.06)	<.001
45-64	0.19 (0.16-0.22)	<.001
≥65	1 [Reference]	
Race/ethnicity		
Non-Hispanic		
White	1 [Reference]	
Black	1.17 (0.96-1.44)	.13
Hispanic	0.63 (0.52-0.78)	<.001
Asian/Pacific Islander	0.66 (0.55-0.79)	<.001
American Indian/Alaska Native/other/mixed race	0.53 (0.40-0.70)	<.001
Comorbidities		
Diabetes	1.40 (1.23-1.60)	<.001
End-stage kidney disease	4.26 (3.14-5.79)	<.001
HIV infection	3.43 (1.93-6.10)	<.001
Prior solid organ transplant	1.49 (0.74-3.01)	.27
History of cancer diagnosis	3.03 (2.67-3.44)	<.001
**Delayed**		
Cohort		
Active TB	1.78 (1.61-1.98)	<.001
Comparison	1 [Reference]	
Sex		
Male	1.18 (1.14-1.22)	<.001
Female	1 [Reference]	
Age, y		
0-24	0.03 (0.02-0.03)	<.001
25-44	0.05 (0.05-0.05)	<.001
45-64	0.17 (0.17-0.18)	<.001
≥65	1 [Reference]	
Race/ethnicity		
Non-Hispanic		
White	1 [Reference]	
Black	1.05 (0.98-1.12)	.15
Hispanic	0.74 (0.70-0.79)	<.001
Asian/Pacific Islander	0.62 (0.58-0.66)	<.001
American Indian/Alaska Native/other/mixed race	0.88 (0.82-0.94)	<.001
Comorbidities		
Diabetes	1.75 (1.68-1.83)	<.001
End-stage kidney disease	4.45 (3.68-5.38)	<.001
HIV infection	2.07 (1.56-2.73)	<.001
Prior solid organ transplant	1.03 (0.74-1.52)	.87
History of cancer diagnosis	1.61 (1.53-1.70)	<.001

Abbreviations: aHR, adjusted hazard ratio; TB, tuberculosis.

^a^ Cox proportional hazards regression model adjusted for sex, age group, race/ethnicity, and selected comorbidities.

**Table 4.** Adjusted YPL Stratified by Select Characteristics Among Patients With Active TB Disease Compared With an Age-, Sex-, and Date of Diagnosis–Matched Cohort of Patients Without Active TB Disease, Kaiser Permanente Northern California, 1997-2017^a^

Variable	YPL (95% CI)			*P* value
	Active TB cohort	Comparison cohort	Difference	
Total	11.5 (10.0 to 18.2)	18.5 (18.2 to 18.8)	−7.0 (−8.4 to −5.5)	<.001
Sex				
Male	13.2 (12.3 to 14.2)	19.1 (18.9 to 19.3)	−5.9 (−6.8 to −5.0)	<.001
Female	8.1 (6.7 to 9.5)	17.3 (17.1 to 17.6)	−9.2 (−11.1 to −7.3)	<.001
Age, y				
0-24	−43.2 (−54.3 to −31.1)	−47.1 (−49.6 to −44.6)	3.9 (−8.4 to 16.2)	.26
25-44	−24.8 (−28.5 to −21.2)	−22.8 (−23.7 to −22.0)	−2.0 (−4.5 to 1.5)	.15
45-64	−2.9 (−4.3 to −1.6)	0.1 (−0.3 to 0.5)	−3.1 (−8.4 to −5.5)	<.001
≥65	22.9 (21.8 to 23.9)	26.0 (25.8 to 26.2)	−3.2 (−4.2 to −2.1)	<.001
Race/ethnicity				
Non-Hispanic				
White	14.0 (12.2 to 15.8)	18.8 (18.6 to 19.1)	−4.9 (−6.5 to −3.1)	<.001
Black	11.2 (8.5 to 13.9)	17.3 (16.6 to 17.9)	−6.1 (−8.8 to −3.4)	<.001
Hispanic	8.4 (6.2 to 10.6)	17.4 (16.9 to 18.0)	−9.0(−11.4 to −6.6)	<.001
Asian/Pacific Islander	11.1 (10.0 to 12.2)	17.7 (17.0 to 18.3)	−6.5 (−7.9 to −5.3)	<.001
American Indian/Alaska Native/other/mixed race	13.1 (10.4 to 15.8)	18.7 (18.0 to 19.4)	−5.6 (−8.8 to −2.4)	<.001
Comorbidities				
Diabetes	10.8 (9.5 to 12.1)	16.4 (16.0 to 16.8)	−5.6 (−7.0 to −4.2)	<.001
End-stage kidney disease	6.8 (3.9 to 9.6)	12.2 (10.3 to 14.1)	−5.5 (−9.3 to −1.6)	.002
HIV infection	1.6 (−3.2 to 6.4)	11.7 (8.7 to 14.8)	−10.1 (−15.8 to −4.4)	<.001
Prior solid organ transplant	7.9 (1.5 to 14.4)	20.4 (16.5 to 24.3)	−12.5 (−21.0 to −4.0)	.002
History of cancer diagnosis	9.9 (8.1 to 11.7)	17.1 (16.6 to 17.6)	−7.2 (−8.8 to −5.6)	<.001

Abbreviations: TB, tuberculosis; YPL, years of potential life.

^a^ YPL compared with United States Life Tables, 2015 with allowance for higher or lower life expectancy than the US census estimate. Linear regression model adjusted for sex, age group, race/ethnicity, and selected comorbidities.

## Discussion

Active TB disease is preventable, but screening and treatment for LTBI is underutilized in the United States.^3^ Engagement in TB prevention by clinicians, health systems, and policy makers is low, partially owing to an underappreciation of the full burden of active TB disease. In this cohort study, we evaluated the risk of mortality among persons with active TB disease in an integrated health system who were insured and had access to high-quality health care. We found that compared with a comparison cohort of KPNC members, persons with active TB disease had an increased risk of early and delayed mortality and significant YPL loss even after adjusting for known factors that increase the risk of developing active TB disease (eg, diabetes, HIV infection, end-stage kidney disease, and solid organ transplantation). Expanding on prior studies, we calculated both the early and delayed mortality risk associated with TB and provide additional data on the potential value of investing in TB prevention.

Patients with active TB disease within KPNC were overall similar to persons with active TB disease in California; a high percentage were Asian/Pacific Islander and 25 years or older, and a substantial percentage had comorbidities that are known to increase risk of TB reactivation, such as HIV infection.^4^ Compared with the comparison cohort, persons with TB were more likely to be Asian/Pacific Islander and, as expected, were more likely to have comorbidities that increase the risk of LTBI reactivation (eg, HIV infection). Although many factors are associated with increased risk of progression to active TB disease in the literature (eg, use of tumor necrosis factor alpha inhibitors),^2,28^ we were only able to include variables that could be extracted from our EHR databases and have high enough prevalence to be meaningfully included in this analysis; for example, we did not include persons who have been exposed to tumor necrosis factor alpha inhibitors, as in the 20-year period included in this analysis, we found only 24 persons with active TB who had been prescribed a tumor necrosis factor alpha inhibitor. Thus, we recognize the inherent limitation of not being able to completely account for all covariates that might meaningfully influence the association between active TB disease and mortality compared with a comparison cohort. However, to our knowledge, our study is the only one to date to assess early and delayed mortality risk adjusting for comorbidities.

Interestingly, we found different factors associated with early vs delayed mortality among persons with active TB disease. For example, HIV infection was only associated with early mortality, whereas diabetes was only associated with delayed mortality (Table 2). This exploratory analysis suggests that certain comorbidities might increase early and delayed mortality risk differentially among patients with TB; this finding should be explored further.

We found increased risk of both early and delayed mortality among patients with active TB disease compared with a comparison cohort after adjusting for age, sex, race/ethnicity, and selected comorbidities (Table 3). Patients in the active TB cohort had an increased aHR in all subgroups compared with the comparison cohort, suggesting that the increased hazard is associated with the early and delayed effects of active TB disease rather than the key comorbidities that increase risk of TB reactivation. This finding suggests that active TB, even when treated, may lead to increased mortality and YPL loss. There are several possible mechanisms for increased delayed mortality. International studies of posttreatment mortality among TB patients suggest high rates of relapse leading to death,^29^ but this does not seem to be the case in the United States^30^ and more specifically in our cohort. Post–TB disease pulmonary sequelae, decreased pulmonary function, and other sequelae have been noted.^9,11,12,14,15,17,18,20^ In addition, persons with some severe forms of TB disease (eg, TB meningitis) have poor survival even after completion of treatment.^19^ Alternatively, among patients with comorbidities, TB disease might complicate management of the underlying comorbidity and thus lead to increased risk of death; for example, drug-drug interactions between TB and antiretroviral medications can lead to use of second-line or alternative agents; modification or reduction in immunosuppression among posttransplant patients to improve TB treatment outcomes can lead to organ rejection. This study was not designed as a cause-of-death study, and thus we cannot delineate a clear cause among those with early or delayed mortality. However, the increased risk of delayed mortality among persons with active TB disease should prompt additional work into better understanding the long-term sequelae of active TB disease. In addition, a re-evaluation of the potential benefits of LTBI screening and treatment may be warranted, especially among persons at high risk of TB reactivation, such as those with immunosuppression, diabetes, or end-stage kidney disease.

### Limitations

This study has several limitations. First, although we tried to account for all variables that might be associated with both an increased risk of developing TB and an increased risk of mortality, we were limited by either inability to extract certain data or insufficient sample size. Although a fully adjusted analysis that includes all variables that might contribute to mortality is ideal, it is not feasible in either our EHR data set or most other data systems. Thus, there might be residual confounding due to medical comorbidities we did not examine in this analysis or other factors, including unmeasured confounding such as due to differences in socioeconomic status. Despite these limitations, our analysis expands on the work of others to adjust for known medical comorbidities. Second, data on country of birth are not systematically captured in our EHR and could not be extracted as an analytic variable. We recognize this is an important variable to consider for the future. Similarly, certain variables, such as smoking, alcohol use, and other substance use are not available for streamlined extraction within our EHR. Third, KPNC members belong to a large, integrated health system and are thus insured; previous analyses comparing KPNC members to the general adult population of Northern California suggest that the 2 populations are similar but have some differences in socioeconomic status.^31^ These differences could impact the generalizability of our results to all of California or other regions of the United States. Fourth, KPNC members have higher life expectancy than the general US population, and thus we used an internal comparison cohort for all comparisons to minimize introducing bias into the analysis.

## Conclusions

In summary, active TB disease was associated with increased early and delayed mortality and more than 7 YPL lost even after adjusting for demographic characteristics and comorbidities. As most active TB disease can be prevented with screening for and treatment of LTBI, these data provide critical estimates to appropriately value LTBI programs. By improving screening practices and early treatment of LTBI, we may be able to reduce much of the early and delayed mortality associated with active TB.

## Notes

Supplement. **eTable.** Cox Regression-Adjusted Mortality per 1000 Person-Years Stratified by Select Characteristics Among Patients With Active Tuberculosis (TB) Disease Compared With an Age, Sex, Date-of-Diagnosis Matched Cohort of Patients Without Active TB Disease, Kaiser Permanente Northern California, 1997-2017
