# Supplementary material for: Association of Mortality and Years of Potential Life Lost With Active Tuberculosis in the United States
Source: JAMA Netw Open. 2020 Sep 23;3(9):e2014481. doi: 10.1001/jamanetworkopen.2020.14481 (PMC7512053; doi:10.1001/jamanetworkopen.2020.14481)
Supplement: Supplement. — eTable. Cox Regression-Adjusted Mortality per 1000 Person-Years Stratified by Select Characteristics Among Patients With Active Tuberculosis (TB) Disease Compared With an Age, Sex, Date-of-Diagnosis Matched Cohort of Patients Without Active TB Disease, Kaiser Permanente Northern California, 1997-2017 [file jamanetwopen-e2014481-s001.pdf]

Supplementary Online Content

Lee-Rodriguez C, Wada PY, Hung YY, Skarbinski J. Association of mortality and years of potential life lost with active turberculosis in the United States. *JAMA Netw Open*. 2020;3(9):e2014481. doi:10.1001/jamanetworkopen.2020.14481

eTable. Cox Regression-Adjusted Mortality per 1000 Person-Years Stratified by Select Characteristics Among Patients With Active Tuberculosis (TB) Disease Compared With an Age, Sex, Date-of-Diagnosis Matched Cohort of Patients Without Active TB Disease, Kaiser Permanente Northern California, 1997-2017

This supplementary material has been provided by the authors to give readers additional information about their work.

© 2020 Lee-Rodriguez C et al. *JAMA Network Open.*

eTable: Cox regression-adjusted mortality per 1000 person-years stratified by select characteristics among patients with active tuberculosis (TB) disease compared with an age, sex, date-of-diagnosis matched cohort of patients without active TB disease, Kaiser Permanente Northern California, 1997-2017

		**Active TB Rate (95% CI)**	**Comparison cohort Rate (95% CI)**	**Adjusted rate ratio (95% CI)**
Total		24.31 (21.98-26.64)	13.44 (13.17-13.70)	1.81 (1.63-1.99)
Sex	Male	29.36 (25.86-32.85)	15.63 (15.25-16.02)	1.88 (1.62-2.11)
	Female	18.45 (15.47-21.44)	10.80 (10.45-11.16)	1.71 (1.43-1.99)
Age in years	0-24	1.97 (0.00-4.22)	1.11 (0.84-1.39)	1.77 (0-4.04)
	25-44	4.98 (3.20-6.77)	2.29 (2.10-2.48)	2.18 (1.39-3.00)
	45-64	24.23 (20.42-28.04)	8.78 (8.43-9.14)	2.76 (2.32-3.21)
	65+	70.56 (61.42-79.69)	40.62 (39.66-41.58)	1.74 (1.51-1.97)
Race/ Ethnicity	White, Not Hispanic	45.61 (56.29-34.92)	16.76 (17.16-16.35)	2.72 (2.08-3.36)
	Black, Not Hispanic	27.52 (36.85-18.19)	13.51 (14.48-12.54)	2.04 (1.34-2.75)
	Hispanic	24.02 (30.26-17.78)	8.80 (9.35-8.25)	2.73 (2.01-3.47)
	Asian/Pacific Islander	21.16 (23.97-18.36)	8.08 (8.61-7.54)	2.62 (2.42-3.02)
	American Indian/ Alaska Native/ Other/mixed race	22.35 (29.45-15.24)	10.04 (10.82-9.25)	2.23 (1.51-3.00)
Comorbidities	Diabetes	40.54 (34.35-46.73)	32.48 (31.05-33.92)	1.25 (1.21-1.29)
	End stage renal disease	106.53 (65.62-147.43)	76.42 (60.34-92.49)	1.39 (1.38-1.41)
	HIV infection	28.81 (12.06-45.57)	18.80 (12.37-25.22)	1.53 (1.52-1.55)
	Post-organ transplant	61.39 (12.73-110.05)	43.72 (25.57-61.88)	1.40 (1.38-1.43)
	History of malignant cancer diagnosis	78.92 (60.00-97.84)	42.58 (40.17-44.98)	1.85 (1.84-1.86)

Cox regression model adjusted for sex, age group, race/ethnicity, and selected comorbidities

P value <0.001 for all rate ratios except age group 0-24 years.

© 2020 Lee-Rodriguez C et al. *JAMA Network Open.*
